# MDD patients with early life stress deactivate the frontostriatal network during facial emotion recognition paradigm: A functional MRI study

**DOI:** 10.1192/j.eurpsy.2022.241

**Published:** 2022-09-01

**Authors:** M. Simon, S. Nagy, Z. Kürtös, G. Perlaki, M. Gálber, B. Czéh

**Affiliations:** 1 Medical School, University of Pécs, Pécs, Hungary, Department Of Psychiatry And Psychotherapy, Pécs, Hungary; 2 Hungarian Academy of Sciences - University of Pécs, Clinical Neuroscience Mr Research Group, Pécs, Hungary; 3 University of Pécs, Szentágothai Research Centre, Neurobiology Of Stress Research Group, Pécs, Hungary

**Keywords:** early life stress, functional MRI, facial emotion recognition, major depressive disorder

## Abstract

**Introduction:**

Early life stress (ELS) is a significant risk factor for major depressive disorder (MDD) in adults. Functional magnetic resonance imaging (fMRI) studies using face emotion processing paradigms have found altered blood-oxygen-level-dependent (BOLD) responses in the cortico-limbic network both in individuals exposed to ELS and in patients with MDD. Thus, early life stress may have a long-lasting impact on brain areas responsible for the processing of socio-affective cues.

**Objectives:**

By applying a facial emotion recognition (FER) fMRI paradigm, we examined the long-term effect of childhood adversity on brain activity in MDD patients with and without ELS.

**Methods:**

MDD patients without ELS (MDD, N=19), those with ELS (MDD+ELS, N=21), and healthy controls (HC, N=21) matched for age, sex, and intelligence quotient underwent fMRI scanning while performing a block design FER task with faces expressing negative emotions. The severity of ELS was assessed with the 28-item Childhood Trauma Questionnaire.

**Results:**

Both MDD and MDD+ELS patients were slightly impaired in recognizing sad faces. Statistical analysis of brain activity found that MDD+ELS patients had significantly reduced negative BOLD responses in the right anterior paracingulate gyrus, subcallosal cortex accumbens compared to HCs. Moreover, the MDD+ELS group had a significantly increased negative BOLD signal in the right postcentral and precentral gyri relative to the HC group. MDD+ELS patients had reduced negative BOLD response in their anterior paracingulate gyrus compared to the MDD group.

**Conclusions:**

Our results support that adult MDD patients with significant ELS are impaired in facial emotion recognition and they display functional alterations in the frontostriatal circuits.

**Disclosure:**

This work was financially supported by the Hungarian Brain Research Program (2017-1.2.1-NKP-2017-00002)

